# Measuring Electrical Activity of the Brain: ERP Mapping in Alcohol Research

**Published:** 1995

**Authors:** David B. Chorlian, Bernice Porjesz, Howard L. Cohen

**Affiliations:** David B. Chorlian, M.S., is a senior scientific programmer in the Neurodynamics Laboratory, State University of New York (SUNY) Health Science Center at Brooklyn, Brooklyn, New York. Bernice Porjesz, Ph.D., is an assistant professor in the Department of Psychiatry, SUNY Health Science Center at Brooklyn, Brooklyn, New York. Howard L. Cohen, Ph.D., is a research scientist in the Neurodynamics Laboratory, SUNY Health Science Center at Brooklyn, Brooklyn, New York

**Keywords:** brain function, brain wave, evoked potential, electrodiagnosis, diagnostic imaging, computer technology

## Abstract

The recording of brain electrical activity from scalp electrodes provides a noninvasive, sensitive measure of brain function. Event-related potentials (ERP’s) are brain waves that are recorded while the subject is exposed to a specific sensory stimulus. Depending on experimental conditions, ERP’s are useful in studying many brain functions, such as sensory and information processing (e.g., memory). The assessment of ERP’s is useful in studying the effects of alcohol on brain function and in identifying people at risk for developing alcoholism. Computerized mapping techniques produce graphs or color-coded images to summarize data about the generation of ERP’s in time and space.

Recording brain electrical activity using scalp electrodes provides noninvasive, sensitive measures of brain function (e.g., cognition). These electrical recordings consist of two phenomena: the continuous electroencephalogram (EEG) and timepoint-specific event-related brain potentials (ERP’s). Unlike positron emission tomography (PET) or magnetic resonance imaging (MRI) techniques, brain mapping does not construct an image of a hidden anatomical structure; rather, it illustrates spatiotemporal brain activity (i.e., brain activity as it occurs in both space and time). This article presents a variety of brain mapping techniques, focusing on the ERP component P300.

## Recording Brain Electrical Activity

The EEG is not linked in time to any specific event but instead reflects the activation level of various brain regions. For example, a person in a relaxed state will manifest a great deal of simultaneous brain wave activity occurring at a wave frequency between 8 and 12 cycles per second. Conversely, ERP’s represent brain electrical activity in response to specific sensory or cognitive events occurring at a specific time. In addition, ERP’s consist of characteristic, highly reproducible wave forms that can be measured to within a fraction of a second, providing an immediate record of the brain activity associated with information processing. Because ERP signals are small and are embedded in the ongoing EEG, statistical techniques are used to extract them from the background EEG.

Depending on experimental conditions, ERP’s may represent overlapping activity of many brain circuits. Therefore, they are useful in studying complex brain functions, such as sensory and information processing (e.g., memory). ERP’s offer a unique approach for assessing brain function because they allow scientists to observe simultaneously electrical activity (i.e., electrophysiology) and cognition.

### How Are ERP’s Obtained?

To record ERP’s, subjects wear a cap embedded with from 20 to 128 noninvasive scalp electrodes. ERP’s can be measured in response to stimuli from any sensory modality (e.g., sight or sound) and also may be recorded while a behavioral task is being conducted. For example, ERP’s can be obtained for visual stimuli using a method in which shapes or letters are presented on a computer screen while the subject performs a simple cognitive task, such as recognizing a particular shape. The subject may be asked to make a behavioral response (e.g., press a button whenever a particular shape appears) or keep count of how many specific stimuli have been presented. Scalp signals reflecting brain electrical activity are recorded simultaneously from all electrodes as the subject processes the stimuli.

### What Do ERP’s Indicate?

The peaks (i.e., positive waves) and troughs (i.e., negative waves) of the ERP wave form, also known as positive and negative components, are measured in microvolts (μV). These components are named according to their positive (P) or negative (N) polarity as well as their latency (i.e., the time of occurrence of the peak wave after the stimulus, measured in milliseconds [ms]). Thus, a negative ERP wave occurring 100 ms following the stimulus would be called the N1 or N100 wave. Early components (with a latency of less than 100 ms) are responses to the physical characteristics of the stimulus (e.g., intensity), whereas the later components are influenced by psychological factors (e.g., cognition).

### What Is a P300?

Much attention has focused on the P300 component of the ERP, a prominent positive component peaking between 300 and 500 ms after a stimulus. Scalp recordings of P300 are strongest at the parietal area, a rear (i.e., posterior) brain region. The ERP task most commonly used to elicit the P300 is the so-called “oddball” task, in which subjects are asked to attend to and/or respond to a rare stimulus presented in a series of other stimuli. For example, in an auditory paradigm, the subject listens to frequent “boops” and rare “beeps” (targets) in a random stream of tone bursts presented about 1.5 seconds apart. The subject may be asked to press a button or count each time a target occurs (for a review, see Regan 1989). P300 results from auditory paradigms, however, are not as consistent as those from visual paradigms. Consequently, the P300 data discussed here are from a study using a visual paradigm in which alcoholics, high-risk subjects, and control subjects pressed a button in response to the rare occurrence of the target letter “X” embedded in a sequence of other visual shapes.

### Advantages of ERP Measurement in Alcoholism Research

ERP’s provide sensitive measures of brain functions. Unlike other imaging techniques, ERP’s reflect subtle, dynamic, real-time, millisecond-to-millisecond transactions that are elicited while the brain is challenged and are therefore highly sensitive to specific brain processes. Most sensory activity occurs within 100 ms after a stimulus is presented, and most cognitive activity takes place within 500 ms. Although images obtained using PET also offer a functional measure of brain processes, electrophysiological measures link images of brain activity to specific timepoints (i.e., provide a temporal resolution) with a precision far greater than that of PET’s. Other imaging methods, such as MRI and computed tomography (CT) scans, portray gross brain structure but do not reflect ongoing electrical activity. Thus, they cannot provide direct measures of brain function. ERP abnormalities, however, can be observed even when MRI and CT images reveal no anatomical changes. In addition, many EEG and ERP characteristics are extremely sensitive to acute and chronic effects of alcohol on the brain and are responsive to intoxication, tolerance, withdrawal, and the effects of prolonged abstinence.

Electrophysiological features also may be hereditary markers of risk for alcoholism. Evidence indicates that the characteristics of both EEG’s and ERP’s are genetically determined and that P300 attributes generally differ between alcoholics and non-alcoholics (Porjesz and Begleiter 1995). A recent twin study (O’Connor et al. 1994) reported that P300 amplitude is highly heritable; similar findings on the heritability of P300 are being obtained by researchers in the Collaborative Study on the Genetics of Alcoholism (COGA) through a large family study (Porjesz et al. in press).

## Methods of ERP Analysis and Mapping

### Amplitude and Latency Measures

**Figure 1:**
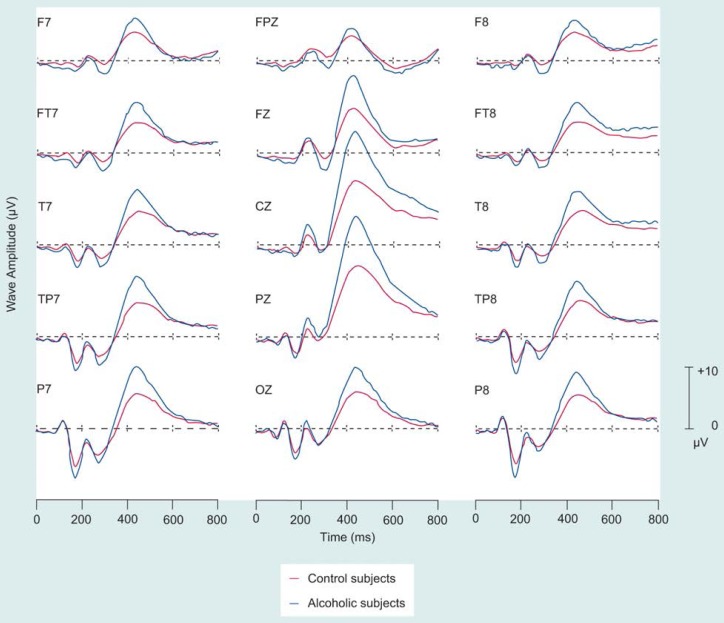
Electroencephalographic (i.e., brain wave) tracings of the waveform P300 obtained from nonalcoholic (i.e., control) and alcoholic subjects in response to a visual stimulus. Each tracing represents a different location on the scalp. The horizontal scales represent time in milliseconds (ms). The vertical scales represent wave amplitude measured in microvolts (μV) of electrical potential. The data are statistically derived from 197 subjects and 61 scalp electrodes.

Researchers have used various auditory and visual paradigms to study the P300 component and have found the P300 amplitude to be smaller in alcoholics than in control subjects (figure 1) (for a review, see Porjesz and Begleiter 1993). Researchers previously thought that these low P300 voltages resulted from neurotoxic effects of alcohol on the brain. Low P300’s, however, do not recover with prolonged abstinence and can occur in subjects at risk for alcoholism prior to alcohol exposure. Thus, low P300 amplitudes may characterize populations at risk for developing alcoholism (Polich et al. 1994).

### Topographic Representations

**Figure 2:**
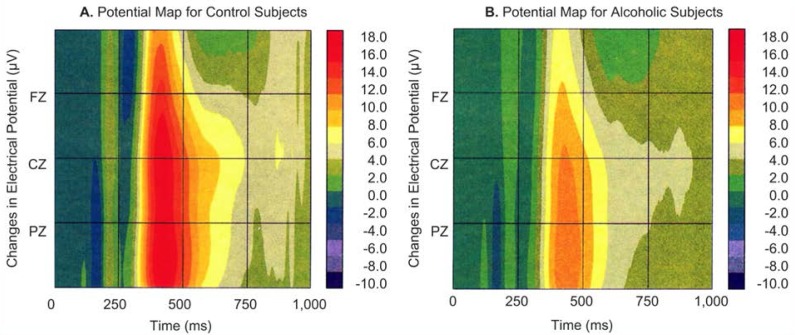
Spatiotemporal (i.e., space-time) potential maps displaying electroencephalographic (i.e., brain wave) tracings of the wave form P300 obtained from (A) nonalcoholic (i.e., control) and (B) alcoholic subjects in response to a visual stimulus. This same data are shown in figure 1. The horizontal scales represent time in milliseconds (ms). The color-coded vertical scales represent changes in electrical potential measured in microvolts (μV) in a spatial continuum along a scalp line from front to rear. Several such maps are needed to demonstrate the spatiotemporal changes at all electrodes. The maps show that although large amplitude differences exist over time in P300 between the nonalcoholic and alcoholic subjects, spatial distributions between the two groups are similar.

**Figure 3:**
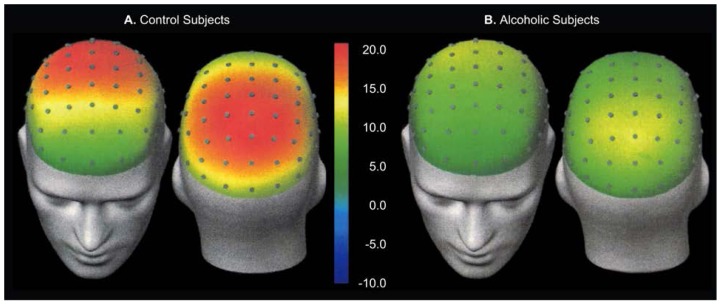
Topographic maps of (A) nonalcoholic (i.e., control) and (B) alcoholic subjects representing electrical potentials (microvolts) recorded 430 milliseconds after exposure to a target stimulus (when P300 is at its maximum). Because the maps indicate fewer electrodes than points, mathematical models must be used to estimate values for the intermediate points. These two maps show little variation in potential.

Although amplitude and latency measures provide important information about brain function, they do not address the spatial distribution of ERP’s across the scalp. Spatial distributions provide information about brain areas possibly involved in generating ERP activity. Displaying ERP’s from multiple electrodes does not show values across the entire scalp surface but only at the sites of the electrodes. Mathematical techniques enable researchers to assign values to the points that have not been directly measured; maps can then be created by calculating the values between electrode points. Figures 2 and 3 show two types of maps—spatiotemporal and topographic—based on this approach (Fein et al. 1992).

Topographic maps are frozen at a single point in time, thereby losing the dynamic aspect of the recorded data. With the advent of computer technology, these spatial maps can be presented as a series of rapidly changing, computer-generated animations that describe how the topographic distribution of the recorded activity changes over time.

## Mapping of Current Source Density

**Figure 4:**
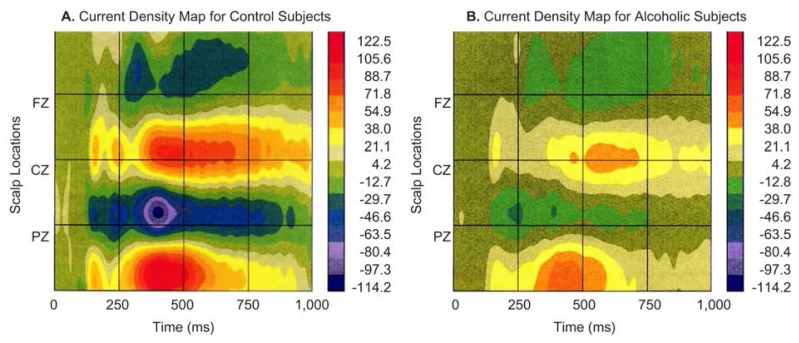
Current source density (CSD) maps are created by taking from all the electrodes simultaneous data and converting them into spatial data. These spatiotemporal (i.e., space-time) maps compare the CSD derived from P300 data of (A) nonalcoholic (i.e., control) and (B) alcoholic subjects. Time is measured in milliseconds (ms). The color-coded vertical scales are proportional to voltage per unit of scalp area.

**Figure 5:**
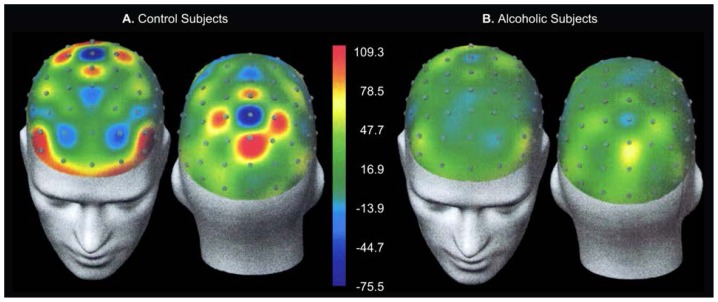
Topographic map of current source density of P300 data from (A) nonalcoholic (i.e., control) and (B) alcoholic subjects 430 milliseconds after exposure to a stimulus. This mapping technique shows local variation, with sources of brain electrical activity more sharply delineated in the control, than in the alcoholic, subjects. The color-coded vertical scale is proportional to voltage per unit of scalp area.

Because voltages measured at a point on the scalp result from both local (i.e., directly under the electrode) and remote sources by mathematically transforming the ERP data, researchers can emphasize local sources. The data transformation produces a quantity known as the current source density (CSD), which represents the radially oriented current density at a point on the scalp. The CSD values denote current sources and “sinks” (i.e., locations where the current becomes undetectable). Researchers create CSD maps by taking simultaneous data from all the electrodes and converting them into spatial data. A CSD topographic map or spatiotemporal map (figures 4 and 5) may display results in a more useful fashion than a topographic map based on ERP’s (figures 2 and 3).

In the P300 experiment mentioned earlier, for example, the peak of the P300 component (occurring at 430 ms) from 61 scalp electrodes is transformed to CSD. In healthy control subjects, these maps indicate both a large posterior focus of activity as well as front (i.e., anterior) foci (figure 4). These brain-mapping techniques aid in pinpointing possible sites of brain dysfunction in alcoholics and in subjects at risk for developing alcoholism.

A comparison of CSD maps obtained from alcoholics and nonalcoholics shows weaker activity in the alcoholics’ brains. Figure 5 displays the distribution of P300 activity across the alcoholics’ scalps. Compared with control subjects, alcoholics can appear to have a weaker posterior focus of activity and no clear frontal focus. Therefore, control subjects and alcoholics exhibit not only differences in amplitude but differences in spatial distributions, particularly in the brain’s frontal regions.

A more elaborate analysis of the electrophysiological data measures information communication from one location in the cerebral cortex to another. If a temporal pattern of electrical activity at one electrode matches the pattern at another electrode a few milliseconds later, information is likely being transferred from the first to the second location. Gevins and colleagues (1990) developed a mathematical method of examining time-lagged CSD data while a subject simultaneously performs a task. The results are interpreted as reflecting the activity of active networks in the cerebral cortex and can be applied to various ERP paradigms.

### Structural Findings

To determine the relationship between brain structure and function, researchers have investigated the neuroanatomical origins of P300 using various techniques. Evidence from recordings obtained from electrodes implanted in the human brain implicates both the medial temporal lobe^1^ as well as source(s) within the frontal lobe as contributing to P300 generation. These findings, coupled with the rather small effect that temporal lobe removal has on scalp P300 during auditory oddball tasks, suggest that multiple brain sites contribute to the P300 (for a review, see Regan 1989).

Researchers have used noninvasive imaging techniques to assess brain structure. In a recent study using P300 amplitude and MRI, Ford and colleagues (1994) reported that reduced P300’s recorded during both automatic and effortful attention tasks (i.e., tasks requiring subjects to focus intently) correlated with frontal and parietal gray-matter volumes. These findings are consistent with the CSD maps of P300 described earlier in which both parietal and frontal foci of activity were found. The reduced P300 amplitudes in alcoholics and their CSD maps may be manifestations of frontal lobe damage (see Oscar-Berman and Hutner 1993).

## Conclusions

Electrophysiological recordings of ERP’s reflect the activation of neural circuits involved in the mediation of sensory and cognitive processes. The assessment of ERP’s has proven extremely useful in studying the effects of alcohol(ism) on brain function and in identifying people at risk for developing alcoholism. In contrast, conventional imaging techniques have helped identify brain structural changes resulting from alcoholism. Electrophysiological brain mapping is the representation of brain functioning in its spatiotemporal dimensions. These electrophysiological techniques provide exquisite temporal resolution of brain processes and have now been enhanced to provide spatial information about possible brain generators of this activity. These novel techniques not only potentially provide a window into the brain’s function but also may contribute simple clinical tools for identifying both the adverse effects of alcohol consumption on the brain and people who may be at risk for alcoholism.

## Footnotes

1 For a definition of this and other technical terms used in this article, see central glossary, pp. 293–295.
